# Socio-Emotional Explorations of Pre-Service Teachers of English as a Foreign Language

**DOI:** 10.11621/pir.2025.0303

**Published:** 2025-09-15

**Authors:** Ayşe Kızıldağ, Özkan Kırmızı

**Affiliations:** a Aksaray University, Turkey; b Karabük University, Turkey

**Keywords:** socio-emotional competence, EFL teacher education, pre-service teachers, practicum, Türkiye

## Abstract

**Background:**

Socio-emotional competence (SEC) is essential in language teaching, where classroom interactions, pedagogical choices, and student engagement are deeply shaped by emotional dynamics. While teacher emotions have been studied quantitatively, little is known about how pre-service EFL teachers develop SEC during a practicum. This gap calls for qualitative inquiry into their emotional experiences and professional growth.

**Objective:**

To probe how an SEC-infused practicum course influences EFL pre-service teachers’ awareness of the role of emotions in English language teaching during a practicum course lasting for 12 weeks in the Turkish context.

**Design:**

Designed as an exploratory case study, the data come from reflective reports, narratives from weekly mentoring meetings, and lesson plans of practice teaching. The study participants are 12 pre-service EFL teachers enrolled in a practicum course during the 2023/24 academic year fall semester.

**Results:**

Incorporating SEC in the practicum processes significantly enhanced pre-service EFL teachers’ ability to recognize, reflect on, and incorporate emotional and social factors into their teaching. This awareness influenced their lesson design, classroom management, and perceptions of English as a global, emotionally charged medium of communication.

**Conclusion:**

Language teacher education programs are advised to emphasize the role of emotions by implementing socio-emotional competency-based principles and practices throughout the curriculum.

## Introduction

A positive classroom atmosphere can be sustained through a few good strategies and emotionally strong teachers. The emotional competence of teachers enables them to establish effective teaching environments with due attention to the affective aspects, reducing teacher burnout and turnover (Benesch, 2017; Jennings & Greenberg, 2009). Theoretically, social and emotional competence (SEC) refers to the integrated ability of teachers to understand, express, and regulate their emotions in ways that foster supportive relationships and promote optimal learning environments (Schonert-Reichl, 2017). SEC involves both intrapersonal skills, such as emotional self-awareness and regulation, and interpersonal skills, such as empathy, perspective-taking, and relational sensitivity (Donahue-Keegan et al., 2019; Zhang et al., 2024). When embedded in teacher education, SEC supports the development of emotional literacy, reflective capacity, and relational responsiveness, all of which enhance the capacities for classroom management, student engagement, and culturally sustaining pedagogy (Oberle & Schonert-Reichl, 2017). Recent studies have consistently shown that teachers with strong SEC are better equipped to manage stress, foster inclusive classroom climates, and engage students more effectively, especially in linguistically and culturally diverse contexts (Siacor et al., 2023). Furthermore, recent intervention-based research demonstrates that cultivating SEC in teachers through mindfulness, coaching, and experiential learning leads to improvements in well-being, relational teaching quality, and resilience under pressure (Brown et al., 2023; Jennings et al., 2019). This body of scholarship positions teachers, including the participating pre-service EFL teachers in this study, not simply as content transmitters but as emotionally attuned practitioners who shape the socio-emotional and academic dynamics of learning environments through their own self-awareness and relational practices. The relevant literature overall highlights the link of SEC to academic performance. Students with strong SEC teachers tend to perform better academically and have better long-term outcomes (Alzahrani et al., 2019; Guo et al., 2023; Hachem et al., 2022).

Though there is substantial scholarly work on teacher emotions and SEC development, until recently there has been a neglect of emotions in Teaching English to Speakers of Other Languages (TESOL) by the dominance of rationality and cognition, as well-stated by White (2018). SEC is significant for language classrooms because communication involves the interaction of people, with their emotions always in the background (Dewaele, 2010). Furthermore, Gkonou and Mercer (2017) emphasize that English teachers are highly engaged with communicative language teaching in classrooms where the focus is understandably on “authentic classroom interactions, peer collaboration, and co-operative pair and group work activities” (p.8). They further remind us that the English language today, in a multilingual world more than ever, requires intercultural competence to move beyond the classroom along with students’ communicative skills. SEC, in this sense, is what both English teachers and their students need to address multilingual backgrounds. A final point is the growing influence of ELF and World Englishes in ELT, which de-centre native-speaker norms and foreground intelligibility, accommodation strategies, and the legitimacy of diverse English varieties. increasing role of English within the EFL context, apart from native competence, as in the English as a Lingua Franca (ELF) and World Englishes (WE) perspectives in the ELT profession. “[I]n the case of a lingua franca, a language is appropriated by outsiders and adapted to suit their own communicative requirements” (Widdowson, 2017, p. 102). How ELT can respond to the global spread of English in terms of classroom practices is a central question (Vettorel & Antonella, 2023). Importantly, addressing this challenge is not only a matter of linguistic strategy but also one of emotional and intercultural sensitivity, areas directly supported by SEC. EFL teachers must guide students in developing empathy, adaptability, and emotional intelligence required for communication across diverse sociolinguistic norms. Thus, SEC becomes essential for preparing both teachers and learners to thrive in emotionally nuanced, globally interconnected English-language classrooms.

When it comes to teacher education programs, it is important to include socio-emotional learning throughout the curriculum and thus, candidate teachers could earn competence before they start the profession (Aspelin, 2019; Kasperski & Hemi, 2022; Waaajid et al., 2013) to equip themselves with the skills for responding to the abovementioned situations. They might reformulate their pedagogical decisions accordingly, and analysis of students’ learning needs and responses to teaching practices can result in appropriate learning interventions, highlighting the importance of evidence-informed decision-making skills (McMillan, 2003). Yet, limited agency and autonomy (De Costa et al., 2018), unsupportive administration, along with little or no collegial support (Pennigton & Richardson, 2016) cause stress and anxiety, which might block appropriate decision-making and thus inhibit positive emotional atmosphere in TESOL (Martinez Aguado, 2018). This is another reason to promote an earlier awareness of SEC competence that necessitates pre-service teacher education programs that take initiatives for building and enabling curricular spaces (Murano et al., 2019; Valente & Lourenço, 2022) for dealing with emotions and enabling pre-service teachers to develop an acute awareness for moving among the complex dynamics of both their and learners’ emotions.

Thus, early SEC awareness and acquisition of competence are also indispensable for further professional development (Palomera et al., 2008). SEC-based teacher education programs enhance culturally responsive teaching by addressing diverse student characteristics effectively (Donahue-Keegan et al., 2019). EFL teacher education, by its nature, requires multicultural interactive pedagogies, since English acts as a global language (De Costa et al., 2018; Kaçar & Bayyurt, 2018).

While SEC has been discussed broadly, its role during pre-service EFL practicum experiences remains underexplored, notably in the Turkish context. Therefore, we attempted to incorporate SEC into a practicum for a group of senior pre-service teachers while practicing in an English Language Teaching (ELT) Department at a public university in Türkiye during the fall semester of the 2023/24 academic year. Student-teachers were assigned to a local middle school for a practicum course, with a university course lecturer as their supervisor and an English language teacher as their mentor. While they were familiarizing themselves with the school norms and staff , such as other subject teachers, school administrators, and the students, they also conducted observations and practice-teaching sessions by reflecting on their professional learning with the mentor. They focused on lesson planning, the use of teaching materials, and overall school management and student relations.

Our aim was to understand the pre-service teachers’ socio-emotional dispositions during their 12-week practicum period. Furthermore, as there was no emphasis on emotions in the teacher education curriculum in question, we designed the study accordingly. Thus, we examine the following research question in this study:

*How does an SEC-integrated practicum course enhance pre-service EFL teachers’ awareness of the role of emotions in ELT?*

## Methods

This research was designed as an exploratory case study (Merriam & Tisdell, 2016). Exploratory case studies are appropriate when researchers investigate aspects of familiar phenomena that remain underexplored in their *local natural* context (Yin, 2014). (Yin, 2014). Through case studies, it is appropriate to investigate a system and understand its underlying principles by enabling researchers to explore a complex phenomenon in-depth from multiple perspectives in a particular setting (Schwandt & Gates, 2018). In this study, we questioned the participants’ explorations of socio-emotional skills to be employed while practicing teaching in the local school they were assigned to. Hence, an exploratory case study would fit our context regarding answering the main research question to understand the impact of a SEC-integrated practicum on the participants’ awareness of the role of emotion in ELT.

### Participants

Seven females and five males participated, aged 22 to 27, enrolled at an EFL teacher education program in a public Turkish university in the fall semester of academic year 2023/34. EFL teacher education departments in Türkiye deliver courses for four years with the content of four-language skill development, applied linguistics, and teaching methodology. In the fourth year, student-teachers register for a practicum course where they spend six hours assigned to a local school, usually either a middle or a high school, with a mentor working at the school as an English-language teacher and a university supervisor from the department. Student-teachers are required to observe their mentors while teaching and then practice teach at local schools for 12weeks. They also have to attend a two-hour mentoring meeting with the supervisor on campus weekly, where they discuss the issues they observe and/or experience during practice-teaching.

The participants in this study were the fourth-year student-teachers who were also enrolled in the practicum course.

All were Turkish, and English was a foreign language for them. The linguistics proficiency levels are between B2 and C1 according to the Common European Framework of Reference for Languages (CEFR), as shown in *Table 1*. They were practice teaching at a local middle school, grades between 5 and 8. The group was already assigned to the first author as their university supervisor to conduct their practicum observations and evaluations and follow their professional progress through weekly mentoring meetings. Thus, a convenient research sampling procedure was followed for the participant recruitment.

**Table 1:** Overview of the Participants

Code	Gender	Age	English Language Proficiency	Practice Teaching Grade
P1	Male	24	B2	7th grade
P2	Male	23	B2	7th grade
P3	Female	22	B2+	6th grade
P4	Male	23	C1	6th grade
P5	Female	26	B2	7th grade
P6	Female	24	B2+	7th grade
P7	Female	24	B2	5th grade
P8	Male	22	B2+	5th grade
P9	Male	23	C1	8th grade
P10	Female	24	B2	8th grade
P11	Female	27	B2	5th grade
P12	Female	25	B2	5th grade

The study was conducted ethically. The participants were invited to the research study at the beginning of the semester; they were informed about the goals of the research and their roles in it. After their oral approval, the consent forms were taken from each by stating that they were free to withdraw from the study at any point if they felt like they could not continue. They also were clearly guaranteed that their identity was not disclosed to third parties in any part of the data. Each participating student-teacher was coded with a number and represented as P1, P2, and so on for documenting narratives in the findings section below.

### Data Production and Analysis

Data were produced in the form of narratives based on the weekly reflective reports from each participant during the 12-week practicum program based on four lesson plans that each participant prepared and taught at the local school they were assigned to, and the discussions from the weekly mentoring meetings on campus with the supervisor. As the practicum supervisor, the first author conducted all the data production. As a result, 144 reflective reports and 48 lesson plans comprised the 943-minute mentoring meeting content. Meetings were audio recorded and transcribed by the first author utilizing a free online application (media.io).

All the qualitative data were manually analyzed via content analysis (Patton, 2015), allowing for an inductive and interpretive approach to explore the participants’ socio-emotional experiences during their practicum. The process began with both authors independently reading and re-reading the transcribed datasets. This immersive reading allowed each researcher to gain a holistic sense of the data and begin preliminary open coding, during which significant statements, emotional expressions, and recurring pedagogical reflections were highlighted.

Subsequently, the researchers generated initial codes that captured key aspects of socio-emotional competence, such as the classroom and the curriculum, to encompass the emotions, teacher-student relational dynamics, and socio-cultural sensitivity. These codes were then organized into broader categories and emerging themes, which reflected patterns across participants’ narratives, such as the emotional impact of the practicum.

To ensure trustworthiness and interpretive alignment, the two authors met regularly to compare coding structures and thematic interpretations. Discrepancies were resolved through reflexive discussion, guided by our shared understanding of SEC theory and qualitative rigor. Where differing interpretations arose, we revisited the data collaboratively and refined the thematic structure until reaching a consensus. This enhanced the credibility of the findings by reducing individual researcher bias (Patton, 2015).

Verbatim excerpts were selected jointly by both authors to illustrate key themes and preserve the authenticity of participants’ voices. Excerpts were chosen based on their representativeness, richness, and relevance to the identified themes. Throughout this process, we maintained an audit trail documenting decisions about coding, theme development, and excerpt selection.

## Results

*Table 2* displays the overall findings. Participants displayed awareness on two levels: emotional and social. While the emotional level relates to student and student-teachers’ emotions regarding in-classroom and out-of-classroom pedagogical issues, the social dimension highlights English’s role as a global lingua franca and its communicative power across diverse contexts. In other words, participants position themselves in relation to both designing the course content and using the English language within the framework of emotions and social contexts.

**Table 2:** Overall Findings

	Themes	Categories	Codes
Emotional dimension	Emotions in the classroom	Classroom as an emotional space	Student emotions Safe emotional space
	Teachers’ emotions as a professional characteristic	Teachers’ own socio- emotional management	Parental involvement Managerial interventions Collegial support
	Emotions in the lesson design/materials	Teaching for and with emotions	Topics involving emotions Emotions in intra- and interpersonal communication
Social dimension	Emotions in communicative English	Intercultural communicative competence	Lesson design for (inter) cultural learning Other speaker norms of English from different cultures (native and non-native contexts)
	Emotions for English as a global language	ELF awareness	WE content/communication Locating EFL within a wider ELT area

## Emotional Dimension

The first theme we found was the emotional dimension. Participants contextualized the classroom as an emotional space where students’ emotions substantially influence instructional decisions. For instance, a participant elaborated on her observations of the student characteristics in the following excerpt:

Excerpt 1:

What I most see in the classrooms is students are so sensitive. They are in puberty, such a critical period for the rest of their lives.… Any word a teacher utters can affect positively or negatively the flow of the course. If the students like the way teachers talk, they can cooperate with her and their friends, and the class can go well. However, from time to time, they might feel inhibited if she is a bit questioning whether they study English at home well enough or whether they care about learning through the course or not. (P6, Reflective report #1)

Participants also developed the issue in the following mentoring meetings with more examples. They exemplified the student emotions with relevance to their personalities and classroom behaviors.

Excerpt 2:

When I was sitting next to a student and observing my mentor teaching, he was not focused on her but me. He was too attentive to who I was, why I was there. He was often peeking into my notes and trying to read my scribbled handwriting. (P2, Mentoring meeting, Week 2)

Excerpt 3:

Sometimes, I think about if students will like me and accept me when I start teaching. I often find myself worrying about my image in their eyes. I am hoping they will find me an interesting and a teacher worthwhile-to-listening to. (P10, Mentoring meeting, Week 3)

It is evident that participants could sense and perceive the students’ emotions in the classroom from the beginning of the practicum. Additionally, P10 in excerpt 3, highlighted the value of students’ emotions on her teaching performance by positioning her professional image as “a teacher worthwhile to listen to” in their eyes. Reflective reports revealed that participants cared for the classroom as a safe space for students’ emotions as well.

Excerpt 4:

I want student would feel secure while speaking of their emotions during my classes. If they feel secure enough to speak out, I believe the classes will be more communicative in nature. (P1, Reflective report #2)

Excerpt 5:

Emotions are seen as a weakness in our society. Being emotional is regarded not valid in most cases. I think it is just the opposite. Once they start to open themselves emotionally, they will feel more confident in speaking their mind in another language. (P8, Reflective report #3)

They also focused on their own emotions and how they were interrelated to issues outside the classroom. One student-teacher (P1) was informed about parental involvement and how it could pose diffculty in his teaching, as he reported in his weekly reflective journal.

Excerpt 6:

I was warned by the mentor that the parents might be quite interfering on how to teach, how much homework is enough and how good a teacher is according to their standards. He gave an example of a parent. She often visited the principal and complained about him because he was assigning too much reading to her son. There start my mentor’s own emotions, and it seems that he can do it well. He sounded like he was used to such interferences. (P1, Reflective Report #2)

In a subsequent mentoring meeting, participants exemplified more similar contexts that could require a teacher’s emotional management. For example, P5 mentioned that clashes might happen between the school management and the teachers regarding some student issues, while another participant, P12, analyzed collegial relations and the emotional imbalances between colleagues.

Excerpt 7:

P5: I experienced an argument between some teachers and the vice-principal about a student in the staffroom. One of the teachers and the vice-principal seemed pretty angry about what students did in one of the classes. Later the students were also invited to the room, and they started to ask questions. Questions like why they did it and whether they understand doing it very disrespectful and irresponsible. Later, some other teachers warned this teacher and the vice-principal that they were too aggressive towards the students and reminded them that the school counselor should have taken the case. Pfff… too much emotion in the room. I felt quite heavy and left .

P12: I was there, too. I think this had to happen in a private room, not in front of others who should not witness. Plus, they had nothing to do with the case, but I was happy that some teachers at least cared for students’ faces. (Mentoring meeting, Week 5)

It is noticeable from excerpts 6 and 7 above that student-teachers perceive the significance of emotions playing a great role between colleagues and administrators. However, P5’s reaction showed that negative emotions might be overwhelming. P12 also revealed her emotional disposition for the situation by taking the side of other teachers who were more sensitive to the students’ emotions and moving the discussion to a private space rather than in front of third parties. P12 would have handled the case differently; she seemed to have made her own decision regarding such conflict management.

The final point where the participants noticed how emotions could play a significant role in their pedagogical decisions was about lesson design and using class materials. While participants were focusing on their lesson plans and reflecting on their choices in the reflective reports and mentoring meetings, one of them delved into the issue of students’ emotional embodiment through her lessons. She illustrated some viable topics that she integrated into her practice teaching.

Excerpt 8:

For example, I chose to plan a lesson on hobbies. I was thinking to elicit their emotional states when they need their hobbies most. Most were game addicts. They stated that they could forget the rest of the world when they were playing on their mobiles. One particular student mentioned gardening with her mom. That was unexpected for me. I asked further questions to understand her point of view. When she was stressed and feeling nervous, she was talking to the plants in their front yard. She stated that she learnt doing it from her mom. (P9, Mentoring meeting, Week 8)

Excerpt 9:

In my teaching session, at the end of a reading text about a scientist’s biography, I asked students to sympathize with the scientist and try to find his feelings while he was working at his age, during the 19th century. Some found his working conditions quite difficult, others exhilarating as he was about to discover something new for humanity. (P3, Reflective report #4)

Overall, the participants demonstrated how the activities in their lesson could elicit learner emotions by tapping into their own emotions in their teaching. They also depicted the individualization of the topics to generate student responses to their teaching. One participant (P11) also mentioned how he utilized an activity to compare emotions between students.

Excerpt 10:

I implemented my lesson plan on different dishes around the world. I asked the students to look at the pictures of the dishes and find out how they might taste. These were the pictures of different cuisines that students have never tried before. I used think-pair-share activity. I wanted them to share their feelings with their deskmates. Then we did a class discussion on their feelings. Some found them delicious, others not quite sure, some others were very conservative, and they would never eat some of the dishes in the pictures. (P11, Mentoring meeting, Week 10)

This theme highlights how pre-service EFL teachers recognized and responded to the emotional dynamics of language classrooms throughout their practicum. Participants conceptualized the classroom as an inherently emotional space where student affect directly influenced engagement, classroom behavior, and instructional decisions. They became increasingly aware of how emotional sensitivity, particularly among adolescents, could shape learning outcomes and teacher-student relationships.

The data also reveal that student-teachers were deeply reflective about their own emotions, often expressing anxiety about being accepted or respected by students. Several participants emphasized the importance of fostering a safe and emotionally supportive classroom climate, challenging cultural assumptions that view emotions as an expression of weakness. They also acknowledged how external factors, such as parental pressure or school leadership conflicts, impacted teacher emotions and professional decision-making.

Participants narrated their awareness of emotions not only in reflective journals, but also applied that awareness in lesson planning and teaching activities. They intentionally integrated emotionally engaging content (*e.g.*, hobbies, biographies, intercultural topics) and promoted emotional expression among students through tasks that encouraged empathy, perspective-taking, and personal reflection. These practices demonstrated how emotional considerations influenced pedagogical choices, classroom interactions, and material design, underscoring the vital role of SEC in shaping responsive, student-centered instruction in EFL contexts.

### Social Dimension

As for the social dimension of participants’ SEC awareness and development, they referred to the position of the English language in the classroom. They decided to make the students aware of the different roles that English could play in students’ future lives. As the study occurred in an EFL context, student-teachers were keen to define how EFL differed from native contexts. Moreover, ELF and WE perspectives were also quite dominant in their lesson planning, explaining how English functions as a global language. Since the current teacher education curriculum highlights these components of English, participants seemed highly aware of the place of English, its different versions and functions in the world. Another theme was the use of English for intercultural communication. They discussed their lesson plans within such perspectives by firmly asserting that such a social dimension must be taught in Turkish schools while students are engaged in English classes.

Excerpt 11:

I prepared a watching task where they could hear different accents from different countries. There was one British and one Italian speaking in a dialogue. In another conversation, a Spanish, a Chinese and an American were speaking on their weekend plans. I asked how they felt after finishing watching the videos a few times. Some students said American speaking was the good English. Others added they could understand Italian but not the Chinese speaker. (P9, Mentoring meeting, Week 11)

In another reflective paper, the same participant reflected on how ELF perspective would free students from the stress of trying to sound like a native speaker. Relaxation on the students’ faces symbolized the participant’s competence in recognizing student emotions about the teaching topic.

Excerpt 12:

I explained the students that good or bad English is relative. I made sure that if they sound comprehensible and convey the intended meaning to the next person, there would be no need to imitate the native accents. In our classes at the university last year, we focused on how outer circle speakers of English outnumber the inner circle. I wanted to share this fact with my students. I saw the relaxation on their faces. (P9, Reflective report #9)

Similarly, participants utilized the English language’s potential to empower students’ communicative competence. One student-teacher mentioned how she had students respond to the speakers in the videos by employing empathic feelings. P5 elaborated on how mainstream classes could be altered by prioritizing learner emotions and reactions to the learning materials.

Excerpt 13:

After we finished watching the video, I instructed the students to produce answers considering characters’ likes for the questions in the video. This is a good way to interact with the characters in the videos. Each student provided their own version and that was fun to do. Creating a light classroom milieu is more enhancing, I observe that they are more willing to engage in the activities, then. (P10, Mentoring meeting, Week 10)

Excerpt 14:

Mostly, classes are mainly based on mechanical activities. I am happy to prove that students could use English for communicating their and others’ emotions. But first changing their thought on that.… My activities were all for this purpose. It will take time, I know, but I feel like it was a great start. (P5, Mentoring meeting, Week 12)

Related to the communicative competence that English classes could provide, a student-teacher referred to locating the students’ own cultural norms and comparing them with the target ones she presented in her lesson plan. It was an activity on different celebrations around the world. The particular focus was springtime and how different countries welcomed the season.

Excerpt 15:

I was surprised how many students were aware of Nauruz. I thought it was a bit outdated in Turkey. It is not celebrated now as it was during my childhood. They stated that they would love to jump over a fire. We also analyzed other cultural celebrations. They outlined the main activities done. They noticed the colorful dresses, delicious food, and festive atmosphere across all celebrations. (P3, Reflective report #10)

In the subsequent week, the participants focused on developing emotions by looking at intercultural competence differently. In a discussion between P3, P5, P9, and P11, some mentioned the rich diversity of cultural themes, whereas others focused on the varieties of English language in different parts of the world.

Excerpt 16:

P3: On one hand, English is a global language. One can expect to see English is everywhere. Yes! But it is not the same language because life is not the same all around. I believe students should notice these differences and I focused on different pronunciation as well as different vocabulary for the same objects. Like elevator and lift , biscuits and cookies.

P5: For me, English being everywhere is more about students’ need for English to communicate with anyone who does not speak Turkish. This is why, I explained to them to speak comprehensibly but not try to be super native-like.

Supervisor: Yes, you both mentioned different perspectives. Thank you. Any other one?

P9: I want these students to find the real chance of speaking with non-Turkish speakers. For me real intercultural learning occurs then. I would love to see the happiness on their faces that they could communicate with them. Oh, EFL context is what makes it limited.

P11: It is a good way to talk about this. If we cannot make time for them to speak to people from other countries, then it is best to teach the culture then. They had a really great time when I did a class on local and traditional clothes. They laughed a lot when they saw men who were wearing turban. They thought this was only for women.

The findings under the social dimension highlight how pre-service EFL teachers became increasingly aware of the global and intercultural role of English and integrated this awareness into their lesson planning and teaching practices. Participants emphasized the importance of raising students’ awareness about the diverse functions and forms of English around the world, differentiating EFL contexts from native-speaking ones and incorporating perspectives from English as a Lingua Franca (ELF) and World Englishes (WE).

They designed tasks to expose students to varieties of English accents and encouraged discussions on the primacy of intelligibility and meaning over native-like pronunciation, thereby reducing students’ language anxiety and fostering emotional reassurance. Student-teachers also promoted intercultural communicative competence by incorporating global cultural themes, such as international celebrations, dress, and vocabulary variation, and by creating classroom discussions that encouraged empathy and emotional engagement with others’ experiences and perspectives.

Moreover, participants critiqued mechanical teaching practices and aimed to create emotionally rich, socially responsive classroom environments that encouraged learners to use English for personal expression, emotional connection, and cultural reflection. Their way of discussing these issues also reflected a shared understanding that English teaching in EFL contexts should prioritize cultural sensitivity, authentic communication, and emotional engagement, all key aspects of developing students’ and teachers’ social and emotional competence.

## Discussion

This study examined a group of pre-service EFL teachers’ awareness and their dispositions regarding SEC. The findings revealed that they could easily recognize the social and emotional dimensions of English teaching in the classrooms through their observation during the practicum. It is evident that participants could connect the content of the previous EFL teacher education program coursework to a real school setting. In this study, we focused on adding SEC for their employment of those skills through their lesson planning and thus analyzing both their and the students’ socio-emotional states. At that point, we witnessed the emergence of noticing emotions in the earlier weeks of the practicum, which was put into action through their practice teaching in the second half of the period.

Considering the classroom as an emotional space, as stated by Sheppard and Levy (2019), empowers teachers to navigate student emotions while teaching; the participants in this study correlated the emotional stability of students with the classroom being a safe space. Fuss and Daniel (2020) proposed that when classrooms are safe spaces, creativity is enabled by being oneself within a non-threatening environment. Another point the student-teachers in the study noticed was collegial emotional support when the school administrators might be misled in conflict resolution. Kaihoi et al. (2022) highlighted the importance of collegial support for teachers’ emotional well-being, in that emotional support is an indicator for reducing professional stress. Emotional well-being is particularly relevant to what student-teachers found a threatening situation, regarding too much and restrictive parental involvement in their professional space.

Another dimension the participating student-teachers observed was how the classroom could be a socio-emotional space for developing intercultural learning, with EFL, ELF, and WE perspectives offering different ways of understanding how English mediates emotions and social relations. The participants’ implementation of practice teaching by the specific choice of content and activity type to elicit student emotions and their awareness of diverse functions of the English language in a variety of contexts across different cultures was revealing for this dimension. The relevant literature points out that EFL classrooms are spaces to explore ELF- and WE-aware language for communication (Bayyurt & Dewey, 2020; Sifakis et al., 2018). As the social-emotional domain of English language teaching involves working with others within an ELF context (Dippold et al., 2019), participants’ pedagogical decisions, focusing on such themes in their practice teaching, might yield appropriate results in their future professional engagements.

A further point is that the study participants adopted their decision-making processes sometimes through their observations, at other times through their school mentor’s advice and/or learning from the previous coursework. For instance, classroom observations during the practicum enabled them to see how emotions were integral to their instructional strategies by closely studying student emotions. Likewise, the school mentors informing them how parents could interfere with classroom teaching was an eye-opener for the participants. In a further case of attempting ELF and WE perspectives with the combination of student emotions, they felt secure, as they had had a few courses discussing such issues in previous years. Elective courses in the curriculum, like *Language and Society* and *Sociolinguistics and Language Teaching,* are mainly based on analyzing linguistic varieties and functions of the English language. Therefore, unlike Deng et al. (2018), who found that the practicum period was full of negative emotions such as anger, anxiety, and shock for pre-service teachers, the participants in our study seemed quite relaxed while sharing their emotions and pedagogical stances freely, far from feeling threatened to reveal their decisions, thanks to the reflexivity that they were engaged in by writing reports and discussing issues during mentoring meetings (Kızıldag, 2023). The reasons are two-fold. One is that the previous coursework was based on critical reflective discussions; the other is that they were quite motivated to become English language teachers in Türkiye, as the profession holds greater prestige than other school subject teaching (Köksal & Ulum, 2018).

## Conclusion

Implementing a socio-emotional perspective in our EFL teacher education was a unique approach, as the program does not include such a standard. Therefore, in an attempt to integrate SEC into the practicum period, we questioned pre-service teachers’ developing awareness of exploiting socio-emotional lesson plans for their involvement in ELT pedagogy. Creating classrooms as emotionally safe spaces, certain choices were dominant in their instructional strategies. Interculturally relevant communicative topics and tasks and combining them with students’ emotions seem to yield highly beneficial results. Additionally, merging ELF- and WE-based activities with students’ emotional states and elevating socio-emotional skills while teaching the English language in the Turkish context were two issues that we found significant in the study.

As for the implications, it is our belief that teacher education programs should explicitly integrate social and emotional competence (SEC) into their curricula to prepare pre-service teachers for the emotional realities of classroom teaching. This includes structured reflection activities that help student-teachers recognize and manage both their own and their students’ emotions. Additionally, fostering intercultural sensitivity and emotionally responsive pedagogy through practical lesson planning tasks can support the development of empathy and communicative competence. Finally, programs should prepare pre-service teachers to navigate emotional challenges related to school dynamics, such as parental pressure and peer conflict, by equipping them with strategies for emotional regulation and professional decisionmaking.

## Limitations

This study has the limitations that a case study naturally embodies. Other studies investigating similar dimensions of the emotional qualities in ELT classes might utilize several qualitative and quantitative research approaches. In addition to this, multi-site studies would yield comparisons and thus reveal context-sensitive hints for examining learner and teacher emotions. Moreover, longitudinal studies might reveal the complex dynamics of the language teachers’ emotional development in professional settings and the changing nature of learner emotions along with their teacher’s SEC engagement. Finally, unlike the short-term research duration employed in this study, further checks could also discover how SEC contributes to emotional regulation for teachers and EFL learners.
